# Occupational contact dermatitis due to captopril

**DOI:** 10.1186/1939-4551-8-S1-A225

**Published:** 2015-04-08

**Authors:** Marisa Rosimeire Ribeiro, Fernanda Komaroff, Laila Sabino Garro, Maria Helena Mattos Porter, Caroline Terumi Adachi, Yara Mello, Maria Teresinha Malheiros

**Affiliations:** 1 Edmundo Vasconcelos Hospital, Brazil

## Background

Approximately 50% of adverse drug reactions to angiotensin-converting enzyme (ACE) inhibitors occur in the skin. While angioedema is a well-known adverse cutaneous reaction to ACE inhibitors, other skin reactions are uncommon. We report the case of a patient with occupational contact dermatitis due to skin contact of the ACE inhibitor captopril.

## Methods

Literature review and case description.

## Results

We assessed a 36 years old female with a history of palpebral and lips oedema, flaking and pruritus, for two months, especially during her work. She worked in pharmaceutical industry and was referred to our outpatient because she had noticed worsening after contact with residues contained in captopril packaging during its manipulation. She had improved when had no contact with the packages. She was treated with topical corticosteroids and oral antihistamines. We performed contact delayed reading test (*patch test*) with captopril in the concentration of 10%, resulting papules, vesicles and swelling at the application site. In addition, there was a negative reaction standard test series. We told her to avoid new exposures to this drug and others with cross reaction.

## Conclusions

Adverse skin reactions to anti-hypersensitive drugs are not uncommon, eczema and rashes usually being caused by thiazides, amiloride or beta-blocking drugs. It is well known that ACE inhibitors elicit angioedema. However, there have been few reports of eczematous skin reactions to ACE inhibitors and all have been due to captopril intake. We reported a patient having occupational contact dermatitis to captopril through skin contact. Although an uncommon event, captopril may elicit eczematous allergic reactions which can be diagnosed by patch-testing.

## Consent

Written informed consent was obtained from the patient for publication of this abstract and any accompanying images. A copy of the written consent is available for review by the Editor of this journal.

